# Effect of Compound Probiotics and Mycotoxin Degradation Enzymes on Alleviating Cytotoxicity of Swine Jejunal Epithelial Cells Induced by Aflatoxin B_1_ and Zearalenone

**DOI:** 10.3390/toxins11010012

**Published:** 2019-01-01

**Authors:** Weiwei Huang, Juan Chang, Ping Wang, Chaoqi Liu, Qingqiang Yin, Andong Song, Tianzeng Gao, Xiaowei Dang, Fushan Lu

**Affiliations:** 1 College of Animal Science and Veterinary Medicine, Henan Agricultural University, Zhengzhou 450002, China; hww5501@stu.henau.edu.cn (W.H.); changjuan2000@henau.edu.cn (J.C.); wangping@henau.edu.cn (P.W.); liuchaoqi2018@stu.henau.edu.cn (C.L.); songandong@henau.edu.cn (A.S.); 2 Henan Guangan Biotechnology Co., Ltd., Zhengzhou 450001, China; gaotianzeng@groundgroup.com; 3 Henan Delin Biological Product Co. Ltd., Xinxiang 453000, China; hndlbio@hndlbio.com; 4 Henan Puai Feed Co. Ltd., Zhoukou 466000, China; lufushan@puaifeed.com

**Keywords:** Mycotoxins, biodegradation, IPEC-J2 cells, probiotics, mycotoxin degradation enzymes

## Abstract

Zearalenone (ZEA) and aflatoxin B_1_ (AFB_1_) are two main kinds of mycotoxins widely existing in grain and animal feed that cause a lot of economic loss and health problems for animals and humans. In order to alleviate the cytotoxic effects of AFB_1_ and ZEA on swine jejunal epithelial cells (IPEC-J2), the combination of a cell-free supernatant of compound probiotics (CFSCP) with mycotoxin degradation enzymes (MDEs) from *Aspergillus oryzae* was tested. The results demonstrated that coexistence of AFB_1_ and ZEA had synergetic toxic effects on cell viability. The cell viability was decreased with mycotoxin concentrations increasing, but increased with incubation time extension. The necrotic cell rates were increased when 40 µg/L AFB_1_ and/or 500 µg/L ZEA were added, but the addition of CFSCP + MDE suppressed the necrotic effects of AFB_1_ + ZEA. The viable cell rates were decreased when AFB_1_ and/or ZEA were added: However, the addition of CFSCP + MDE recovered them. The relative mRNA abundances of *Bcl-2*, *occludin*, and *ZO-1* genes were significantly upregulated, while *Bax*, *caspase-3*, *GLUT2*, *ASCT2*, *PepT1*, and *IL6* genes were significantly downregulated by CFSCP + MDE addition, compared to the groups containing 40 µg/L AFB_1_ and 500 µg/L ZEA. This research provided an effective strategy in alleviating mycotoxin cytotoxicity and keeping normal intestinal cell structure and animal health.

## 1. Introduction

Mycotoxins are ubiquitous and accessible in grain, feedstuffs, human foods, animal products, and soil, and are the secondary metabolites of molds such as *Aspergillus*, *Penicillium*, and *Fusarium*. Presently, about 300 fungal metabolites from more than 100 species of fungi have been reported to have toxigenic potential [1]. Mycotoxins bring a critical threat to food safety due to their adverse impacts on human and animal health [2]. Among many kinds of mycotoxins, aflatoxin B_1_ (AFB_1_) and zearalenone (ZEA) are recognized as the common and major mycotoxin contaminants in agricultural products and their byproducts. AFB_1_ is the most lethal mycotoxin, demonstrating mutagenic, hepatotoxic, carcinogenic, and teratogenic impacts on many kinds of animals: Therefore, it is classified as the top carcinogen [3]. ZEA has been regarded as a kind of nonsteroidal estrogenic compound, causing anabolic activity and hyperestrogenism in the reproductive organs of animals [4]. ZEA is regularly found in corn and its derived byproducts, secreted by *Fusarium graminearum* and other microbes, and has a macrocyclic lactone to bind affinity to estrogen receptors for causing estrogenic effects on pigs [5]. It has also been regarded as a causative agent of infertility in decreasing milk production in cattle [6]. Therefore, mycotoxin contamination is a major health concern for animals and human beings. The current problem is that health risk assessments usually depend on one single mycotoxin, which may neglect the superimposition or competitive interactions among the coexisting mycotoxins [7]. It should be noted that more than one kind of mycotoxin may occur in a given sample. The reasons are that one species of molds may produce more than one kind of mycotoxins or the different samples from different places may contain different kinds of mycotoxins. Thus, the study of the synchronous degradation of both AFB_1_ and ZEA has become more and more important.

Mycotoxin contamination in food and feed samples is a serious recurring problem around the world. A survey was conducted to detect the coexistence of AFB_1_ and ZEA in animal and agriculture products: The detection rates of AFB_1_/ZEA were 65%/49%, 8%/52%, and 50%/19% in Southeast Asia, North America, and Southern Europe, respectively [8]. According to the estimates conducted by the Food and Agriculture Organization, about 25% of worldwide crops are contaminated by mycotoxins, causing economic losses of up to billions of dollars [9]. Therefore, it is important to master mycotoxin levels in feed ingredients to keep animal health and ensure human food safety [10]. 

In order to degrade mycotoxins, many physical and chemical detoxification methods have been developed to inhibit mycotoxigenic fungal growth or remove mycotoxin contamination, but few methods can meet the requirements due to biosafety risks, high costs, or limited binding capacity. It is necessary to find optimal biological detoxification methods to guarantee food safety for animals and human beings [11]. Many species of bacteria, molds, and yeasts have shown the capability to biodegrade mycotoxins. For example, *Mycobacterium fluoranthenivorans*, *Rhodococcus erythropolis*, and other microbes have been reported to pose the aflatoxin detoxifizyme or to have potential for aflatoxin degradation [12,13,14,15,16]. Other research has indicated that *Pseudomonas* sp., *Rhodococcus pyridinivorans*, and *Saccharomyces cerevisiae* are able to degrade ZEA [17,18,19]. Zuo et al. reported that the cooperation of both AFB_1_ degradation enzyme and probiotics could degrade AFB_1_ effectively [20]. Our previous research showed that the combination of mycotoxin degradation enzymes and probiotics were effective for AFB_1_ and ZEA synchronous degradations [21]. 

Interest in biological detoxification of AFB_1_ or ZEA has greatly increased during the past decade. It was reported that *Lactobacillus plantarum* could alleviate disturbances in intestinal DNA fragmentation and gene expressions in mice treated with AFB_1_ or AFM_1_ [22]. Several studies have revealed that lactic acid bacteria and *Bifidobacteria* are able to bind mycotoxins and reduce their toxicity [23,24]. Therefore, the use of probiotics is beneficial to human beings and animals chronically exposed to mycotoxins.

Generally, the intestine acts as the first physical barrier in regulating nutrient and water uptake and excluding potential pathogens and toxicants [25,26,27]. The small intestine usually contacts and absorbs ZEA and AFB_1_ first: Consequently, it is exposed to high mycotoxin concentrations, which certainly affect intestinal health [28]. It has been proven that epithelial cell functions and integrity are disrupted by ZEA [29]. However, studies of inflammatory response, barrier function, and nutrient absorption in the intestinal tract induced by the individual or superimposed cytotoxicity of ZEA and AFB_1_ are still limited.

IPEC-J2, a jejunal epithelial cell line of porcine, is a good model to study the human intestinal immune system and toxin interactions with gut mucosa [30,31]. It has also been used as an initial tool to screen potential probiotic microorganisms for their adhesiveness and anti-inflammatory properties [32]. Intestinal epithelial cells allow nutrient absorption and prevent the passage of pathogens and toxins into systemic circulation. Previous research has indicated that probiotics can protect intestinal epithelial barrier from pathogenic bacteria and mycotoxins [33]. However, the molecular mechanisms of probiotics and the mycotoxin degradation enzymes involved in mycotoxin biodegradation and gastrointestinal tract protection against mycotoxin attacks are unknown.

The objective of this research was to determine the combinatorial toxicity of AFB_1_ and ZEA in vitro, and to reveal the effect of the combination of a cell-free supernatant of compound probiotics (CFSCP) with mycotoxin degradation enzymes (MDEs) on alleviating IPEC-J2 damage induced by ZEA and AFB_1_.

## 2. Results

### 2.1. The Preliminary Reaction Time, Concentrations, and Relationship of AFB_1_ and ZEA Determined by the Exposed IPEC-J2 Cell Viability

Table 1 demonstrates that there were superimposition interactions between AFB_1_ and ZEA in inhibiting cell viability, which was higher than the individual mycotoxin (*p* < 0.05). The relative cell viability was reduced by 7.89% when ZEA + AFB_1_ concentrations were increased from 500 + 40 µg/L to 1000 + 80 µg/L after 24 h reaction (*p* < 0.05): However, it was increased by 16.71% when the reaction time was extended from 6 h to 48 h with ZEA + AFB_1_ concentrations at 500 + 40 µg/L (*p* < 0.05). Single CFSCP + MDE addition had no significant effect on relative cell viability from 6 h to 48 h incubation (*p* > 0.05). After considering the above results and international standards of mycotoxin thresholds in feedstuffs, the optimal reaction conditions for the further cytotoxic experiment were confirmed as 40 µg/L AFB_1_ and 500 µg/L ZEA with 24 h reaction.

### 2.2. Effects of CFSCP + MDE on Alleviating Cell Necrosis and Apoptosis Induced by AFB_1_ and ZEA

Table 2 shows that the necrotic cell rates (Q1), the late apoptotic cell rates (Q2), the early apoptotic cell rates (Q3), and the viable cell rates (Q4) in AFB_1_ and/or ZEA, single CFSCP + MDE, and AFB_1_ + ZEA + CFSCP + MDE groups were significantly different, compared to the control group (*p* < 0.05). The early and late apoptotic cell rates in the A40, Z500 + A40, single CFSCP + MDE, and Z500 + A40 + CFSCP + MDE groups were higher than Z500 and the control groups (*p* < 0.05). It was also found that single CFSCP + MDE addition without mycotoxins could increase necrotic and apoptotic cell rates (*p* < 0.05) and decrease viable cell rates (*p* < 0.05) compared to the control group. However, CFSCP + MDE addition together with AFB_1_ + ZEA were able to decrease necrotic cell rates by 42.69% (*p* < 0.05) and increase viable cell rates by 3.11% (*p* < 0.05) compared to the AFB_1_ + ZEA group without the CFSCP + MDE addition. It could be summarized that a CFSCP + MDE addition in Z500 + A40 had the ability to alleviate mycotoxin cytotoxicity and make necrotic and viable cell rates recover to the same levels as the single CFSCP + MDE addition group. 

### 2.3. Effects of CFSCP + MDE on Inflammation, Apoptosis, Tight Junction (TJ) Proteins, and Nutrient Transport Gene mRNA Abundances of IPEC-J2 Cells Induced By AFB_1_ And ZEA

**Figure 1:**
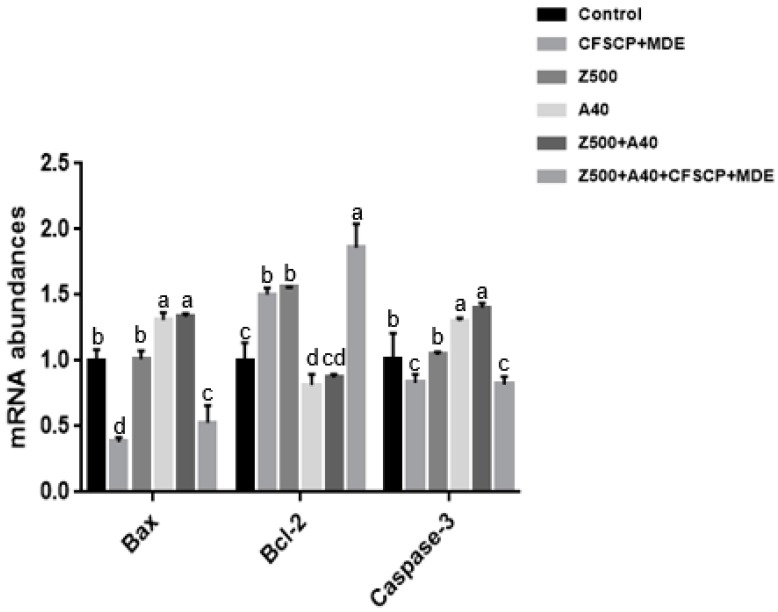
Effects of CFSCP + MDE on mRNA abundances of *caspase-3*, *Bcl-2*, and *Bax* of IPEC-J2 cells induced by AFB_1_ and ZEA for 24 h. Note: The marked different letters on the bars significantly differ from each other (*p* < 0.05), while the same letters insignificantly differ from each other (*p* > 0.05) (the same as below).

Compared to the Z500 + A40 group, the relative mRNA abundance of B-cell lymphoma-2 (*Bcl-2*) was significantly upregulated, while *Bax* and cysteinyl aspartate specific protease (*caspase-3*) were downregulated in Z500 + A40 + CFSCP + MDE and single CFSCP + MDE groups (*p* < 0.05), indicating that CFSCP + MDE was able to alleviate mycotoxin cytotoxicity through decreasing cell apoptosis. The relative mRNA abundances of *Bax* and *caspase-3* were significantly upregulated, while the relative mRNA abundances of *Bcl-2* were downregulated in the A40 and Z500 + A40 groups, compared to other groups (*p* < 0.05) (see Figure 1). 

**Figure 2:**
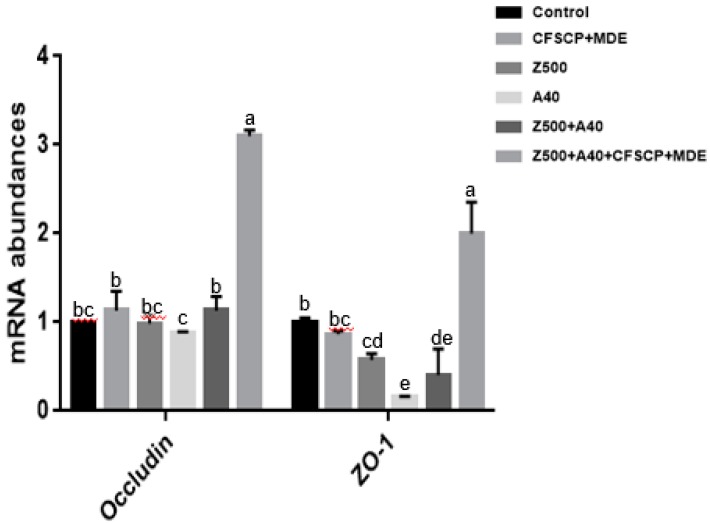
Effects of CFSCP + MDE on relative mRNA abundances of *occludin* and *ZO-1* in IPEC-J2 cells induced by AFB_1_ and ZEA for 24 h. Note: The marked different letters on the bars significantly differ from each other (*p* < 0.05), while the same letters insignificantly differ from each other (*p* > 0.05) (the same as below).

Figure 2 shows that the relative mRNA abundances of occludin and *ZO-1* in the Z500 + A40 + CFSCP + MDE group were upregulated (*p* < 0.05) compared to other groups. The relative mRNA abundance of *occludin* in the A40 group was significantly lower than the Z500 + A40 and Z500 + A40 + CFSCP + MDE groups (*p* < 0.05). The relative mRNA abundances of *ZO-1* in the A40 and Z500 + A40 groups were significantly lower than those in the other three groups, except for the Z500 group (*p* < 0.05). This implied that a CFSCP + MDE addition could make cell structure tight and protect intestinal cells from mycotoxin attack, especially when mycotoxins existed.

**Figure 3:**
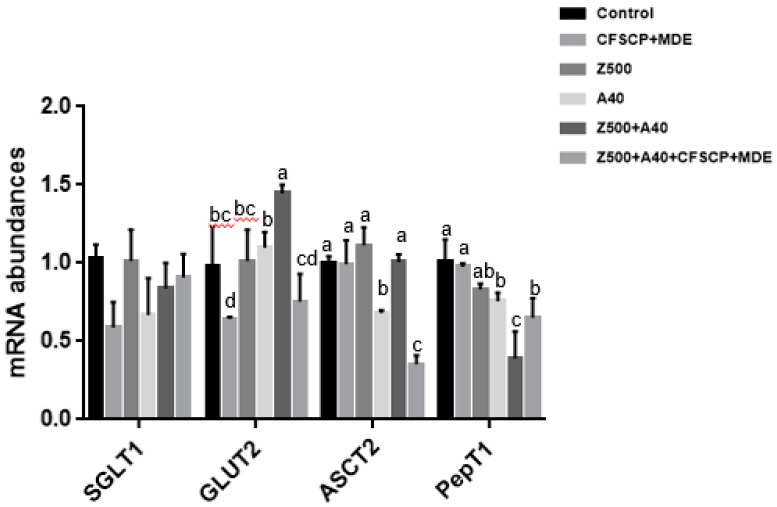
Effects of CFSCP + MDE on mRNA abundances of *SGLT1*, *GLUT2*, *ASCT2*, and *PepT1* of IPEC-J2 cells induced by AFB_1_ and ZEA for 24 h. Note: The marked different letters on the bars significantly differ from each other (*p* < 0.05), while the same letters insignificantly differ from each other (*p* > 0.05) (the same as below).

Figure 3 shows that mRNA abundances of ASC amino acid transporter 2 (*ASCT2*) in the Z500 + A40 + CFSCP + MDE group was significantly downregulated compared to the other groups (*p* < 0.05). The mRNA abundance of facilitated glucose transporter (*GLUT2*) was significantly upregulated by Z500 + A40 and downregulated by the single CFSCP + MDE addition (*p* < 0.05): However, Z500 + A40 + CFSCP + MDE could adjust *GLUT2* to the normal level as the control group to keep regular nutrient transportation. The mRNA abundances of peptide transporter 1 (*PepT1*) in the three AFB_1_-containing groups were significantly lower than the control and single CFSCP + MDE addition groups (*p* < 0.05). CFSCP + MDE addition in Z500 + A40 could upregulate *PepT1* mRNA abundance, compared to the Z500 + A40 group (*p* < 0.05). There were no significant differences for sodium-dependent glucose cotransporter 1 (*SGLT1*) among the six groups (*p* > 0.05). 

**Figure 4:**
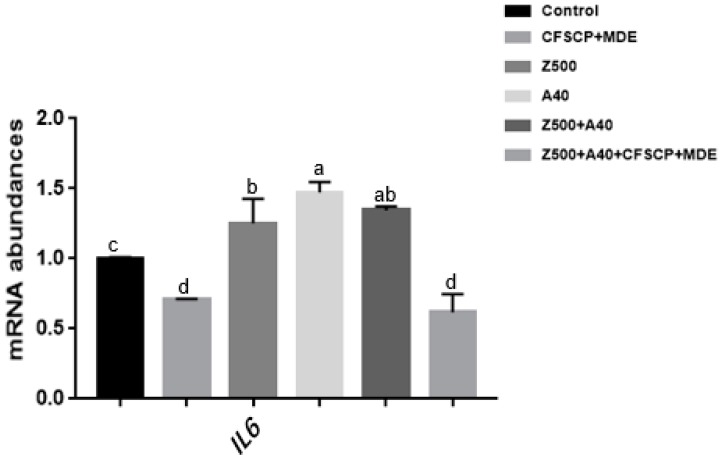
Effects of CFSCP + MDE on interleukin-6 (*IL6*) mRNA abundances of IPEC-J2 cells induced by AFB_1_ and ZEA for 24 h. Note: The marked different letters on the bars significantly differ from each other (*p* < 0.05), while the same letters insignificantly differ from each other (*p* > 0.05) (the same as below).

Figure 4 indicates that AFB_1_ and ZEA alone or together significantly upregulated the interleukin-6 (*IL6*) mRNA expression level (*p* < 0.05): However, single CFSCP + MDE or CFSCP + MDE Z500 + A40 significantly downregulated the *IL6* mRNA expression level (*p* < 0.05). This led to a hypothesis that CFSCP + MDE could alleviate mycotoxin cytotoxicity to decrease inflammation and cell apoptosis. 

## 3. Discussion

Table 1 shows that the coexistence of AFB_1_ and ZEA was more toxic than the individual mycotoxin in inhibiting cell viability at different reaction times and different mycotoxin concentrations. The reason was due to the toxic superimposition of AFB_1_ and ZEA, in agreement with a previous report in which it was proven that the combined cytotoxic effects for kidney, liver, and other cell lines appeared when more than one kind of mycotoxin coexisted [34]. Of course, cell viability is affected by many factors, such as cell type, exposure period, mycotoxin doses and kinds, and metabolites [35]. Why is cell viability increased with reaction time extension? The main reason may be due to the increasing adaptability of intestinal cells under long-time exposure to mycotoxins [35].

When AFB_1_ and/or ZEA was added, the necrotic cell rates were increased, and viable cell rates were decreased. However, a CFSCP + MDE addition could alleviate the toxicity of AFB_1_ and ZEA, which may be from mycotoxin biodegradation [21]. A relevant report indicated that *Lactobacillus acidophilus* and *Lactobacillus reuteri* could inhibit ochratoxin A (OTA) effects on IL-10 and TNF-α secretion and apoptotic induction in the mononuclear cells of human peripheral blood [36]. The early and late apoptotic cell rates were significantly enhanced in the groups containing individual AFB_1_ or AFB_1_ + ZEA, but only ZEA could not induce cell apoptosis, indicating that AFB_1_ is more toxic than ZEA for inducing cell apoptosis. This result corresponds with previous research in which 10–40 μM ZEA could not induce apoptosis in porcine kidney 15 cells [34], but is inconsistent with another research project in which 120 μM (approximately 38.20 μg/mL) ZEA induced a 61.8% apoptotic rate of porcine granulosa cells [37]. The different mycotoxin cytotoxicity may be related to different mycotoxin concentrations and cell types, since the different kinds of mycotoxins have different effects on the various organs [36,38].

Generally, cell apoptosis is inhibited by *Bcl-2* and promoted by *Bax* and *caspase-3* gene expressions. A previous report showed that probiotics could upregulate anti-apoptotic genes such as *Bcl-2* and downregulate apoptotic genes such as *Bax* and *caspase-3* [39]. An AFB_1_ or AFB_1_ + ZEA addition in this study upregulated *caspase-3* and *Bax* mRNA abundance. *Caspase-3* is an important apoptotic biomarker that can be enhanced by various stress-inducing factors [40], indicating that mycotoxins cause cell apoptosis. It was found that the single CFSCP + MDE addition or CFSCP + MDE + A40 + Z500 could upregulate *Bcl-2* mRNA abundance and downregulate *Bax* and *caspase-3* mRNA abundances: It can be inferred that CFSCP + MDE was able to decrease cell apoptosis, consistent with the result measured by the fluoresceine isothiocyanate (FITC) method in this research.

*SGLT1* plays an important role in the small intestine in active glucose uptake, while *GLUT2* helps to diffusively transport intracellular glucose into the bloodstream. *GLUT2* mRNA abundance was upregulated by 500 µg/L ZEA plus 40 µg/L AFB_1_, but it was downregulated to the normal level by CFSCP + MDE addition, indicating that CFSCP + MDE could regulate *GLUT2* gene expression in keeping regular nutrient transport and absorption in the intestines. It has been reported that deoxynivalenol and other mycotoxins affect nutrient absorption in human and mouse intestinal epithelial cells [41,42], in agreement with the mRNA abundance change of the relative gene induced by mycotoxins in this study. *ASCT2* is an amino acid transporter, and is highly expressed in cancer cells [43]: The downregulation of the *ASCT2* gene by a CFSCP + MDE addition will help to alleviate cytotoxicity induced by AFB_1_ and ZEA. *PepT1* is a peptide carrier [44], and the downregulation of the *PepT1* gene by an A40 + Z500 addition may affect peptide metabolism in the intestine. A CFSCP + MDE addition in A40 + Z500 can upregulate the *PepT1* gene to recover normal peptide metabolism.

TJ proteins that are multiprotein complexes mainly sustain the intestinal epithelial barrier [45]. Intestinal tight junctions are the therapeutic target for the modulation of intestinal barrier function and the prevention of various gastrointestinal diseases, including the symposiums caused by mycotoxins. TJ proteins include ZO-1, occludin, and claudin [46]. This result showed that a CFSCP + MDE addition in A40 + Z500 could upregulate the relative mRNA abundances of *ZO-1* and *occludin* in protecting the gut mucosal barrier from damage induced by mycotoxins. The reason may be due to probiotic functions in protecting the intestine from mycotoxin damage. Previous research has reported that *ZO-1* expression of IPEC-J2 cells treated with *Bacillus subtilis* was upregulated against deoxynivalenol-induced damage [47], and that a probiotic mixture could protect the epithelial barrier of mice by maintaining tight junction protein expression and preventing cell apoptosis [48], corresponding with this research. Compared to the single CFSCP + MDE addition, Z500 + A40 + CFSCP + MDE significantly upregulated *ZO-1* and *occludin* mRNA abundances, maybe due to the stimulating response from mycotoxins.

Cytokines are the key signals in the intestinal immune system and play pivotal roles in host defense, inflammatory responses, and autoimmune disease. *IL-6* is a main signal factor of the gut immune and inflammatory response, and is excreted by different stimulators, such as mycotoxins, microbial infections, and other factors [49]. AFB_1_ and ZEA alone or together led to an upregulation of *IL-6* mRNA abundance, indicating that inflammation was triggered by mycotoxins. However, single CFSCP + MDE addition or Z500 + A40 + CFSCP + MDE could downregulate *IL-6* mRNA abundance: It could be inferred that the combination of probiotics and mycotoxin degradation enzymes was able to alleviate the cytotoxicity of mycotoxins. 

## 4. Conclusions

This study demonstrated that the combination of compound probiotics and mycotoxin degradation enzymes could alleviate cell damage, necrosis, inflammatory responses of IPEC-J2 cells induced by ZEA and AFB_1_ through positively regulating gene expressions relative to intestinal cell inflammation, apoptosis, TJ proteins, nutrient transport, and absorption. It provided a new method for alleviating mycotoxin cytotoxicity in protecting intestinal cells from mycotoxin attack.

## 5. Materials and Methods

### 5.1. Chemicals and Cell Culture

AFB_1_ and ZEA were purchased from Sigma-Aldrich (St. Louis, MO, U.S.) and dissolved in dimethyl sulfoxide (DMSO) (Shanghai Solarbio Biotechnology Co., Ltd. Shanghai, China) and 99.6% ethanol to prepare the concentrations of 1 mg/mL and 10 mg/mL as stock solutions, respectively. Work solutions were diluted with Dulbecco’s Modified Eagle Medium/Nutrient Mixture F-12 (DMEM/F12 at 1/1) (Hyclone, Logan, UT, U.S.) without serum and antibiotics. Phosphate-buffered saline (PBS) and thiazolyl blue tetrazolium bromide (MTT) were purchased from Solarbio (Shanghai Solarbio Biotechnology Co., Ltd. Shanghai, China). The final concentrations of DMSO and ethanol used as solvents in the cell culture medium were less than 0.1%. IPEC-J2 cells for subculture use were cultured in DMEM/F12 added with 10% fetal bovine serum (Biological Industries, Kibbutz Beit-Haemek, Israel) without antibiotics. The cells were routinely seeded at a density of 5 × 10^5^ in plastic tissue culture flasks (25 cm^2^), kept in a humidified incubator at 37 °C with 5% CO_2_, and passaged twice weekly.

### 5.2. Microbial Preparation

*B. subtilis*, *Lactobacillus casein* (*L. casein*), *Candida utilis* (*C. utilis*), and *Aspergillus oryzae* (*A. oryzae*) were obtained from China General Microbiological Culture Collection Center (CGMCC). *B. subtilis*, *L. casein*, and *C. utilis* were inoculated in LB, MRS, and YPD media, respectively, and incubated according to the previous report [21]. The above microbes were harvested after 24 h culture for further use. The visible microbial counts were respectively adjusted to 1.0 × 10^7^ count-formed units (cfu)/mL with DMEM/F12. CFSCP was prepared by the following: The compound probiotics were mixed at a ratio of 1:1:1 [21], centrifuged at 4 °C and 12000× *g* for 10 min, and filtered through 0.22 μm Minisart high-flow filter (Sartorius Stedim Biotech Gmbh, Goettingen, Germany) to remove the microbes.

The mycotoxin degradation enzymes for AFB_1_ and ZEA detoxification were extracted from solid-state fermentation of *A. oryzae* according to the following process: The solid-state fermentation was conducted in 250-mL Erlenmeyer flasks containing 15 g of medium, which consisted of corn meal, soybean meal, and wheat bran at a ratio of 1:2:7 (*w*/*w*). After 9 mL distilled water was added, they were homogeneously mixed and autoclaved. About 2 mL of *A. oryzae* at 1 × 10^8^ spores/mL was added to the sterilized medium, mixed, and incubated at 30 °C for 7 d. In order to extract the crud enzymes, the fermented stuffs were mixed with 75 mL DMEM/F12 (*w*/*v*) in a flask, shaken at 30 °C and 150 rpm for 1 h on a rotor wheel, and then stood for 1 h. The mixture was filtered by eight-layer gauze, followed by filtering through Whatman No.4 filter paper (20–25 μm pore diameters), and was centrifuged for 15 min at 3,000× *g* to remove the residue. The crude enzymes were obtained by filtering through a 0.22-μm Minisart high-flow filter to remove the microbes. At last, the MDE from *A. oryzae* was diluted by 320 folds with DMEM/F12 and stored at 4 °C for further use. AFB_1_-degrading enzyme activity (284.3 U/L) was measured with the previous protocol [50] and modified as the following: One unit of enzyme activity was defined to degrade 1 ng AFB_1_ per min at 37 °C and pH 8.0. The ZEA-degrading enzyme activity (31.0 U/L) was measured using the above method modified with ZEA as the substrate.

### 5.3. Experimental Design

In order to determine the effect of CFSCP + MDE on alleviating IPEC-J2 cell damage induced by AFB_1_ and ZEA, the experimental design was as following:

(1) Control group;
(2) CFSCP + MDE;
(3) Toxin group added with 500 or 1000 µg/L ZEA (Z500 or Z1000);
(4) Toxin group added with 40 or 80 µg/L AFB_1_ (A40 or A80);
(5) Toxin group added with 500 µg/L ZEA and 40 µg/L AFB_1_ or 1000 µg/L ZEA and 80 µg/L AFB_1_ (Z500 + A40 or Z1000 + A80);
(6) Toxin group added with 500 µg/L ZEA and 40 µg/L AFB_1_ plus CFSCP + MDE (Z500 + A40 + CFSCP + MDE).

About 3 µL CFSCP and 2 µL MDE were added into the total 100 µL reaction volume at a ratio of 3:2, as in our previous report [21].

### 5.4. Cell Viability Determination

Cell viability was determined by the previous MTT assay [51]. IPEC-J2 cells were seeded at 4000 cells/well in 96-well tissue culture plates (COSTAR, Corning, NY, U.S.) in the cytotoxic experiment. After the cells were adhered for 24 h at 37 °C and 5% CO_2_, the supernatants were discarded. The cells were washed with PBS 3 times, and then 100 μL DMEM/F12 containing the different ingredients without serum and antibiotics in 6 groups was added to the plates according to the above design. The cells were exposed to ZEA (500 and 1000 µg/L) and/or AFB_1_ (40 and 80 µg/L) for 24 h. At the end of the treatments, each well was added with 10 μL MTT solution (5 mg/mL in PBS), and 4 h later the medium was discarded. Thereafter, 150 μL DMSO was added to each well, and the plates were shaken for 10 min to dissolve the purple formazan crystals. Absorbance (A) was measured at 490 nm (A490) and 630 nm (A630) by using an ELx 800 microplate reader (BIO-TEK Instruments Inc., Winooski, VT, U.S.). Cell counting was conducted using a hemacytometer. The relative viability of the IPEC-J2 cells treated with mycotoxins was expressed as the ratio of optical density (OD), compared to the control group.

### 5.5. Determination of Cell Status

IPEC-J2 cells were incubated with AFB_1_ and/or ZEA for 24 h with or without CFSCP and MDE addition, and then harvested. Each well was washed twice with 1 mL PBS for removing the dead cells caused by the trypsin (without EDTA) treatment or cultivation process. Thereafter, 100 μL aliquots (10^5^ cells) of the solutions were put into 1-mL centrifuge tubes and centrifuged at 800 rpm and 4 °C for 5 min to remove the mycotoxins and cellular debris, and the pellets were suspended in 1 mL PBS again. Then, 10 μL propidium iodide (PI) and 5 μL Annexin V–fluorescein isothiocyanate (FITC) were added into the tubes and gently rotated. The cells were incubated at room temperature in the dark for 15 min, and then 400 μL of binding buffer was added to each tube. At last, the cells were measured with flow cytometry (Em 530 nm, Ex 488 nm, Becton Dickinson Company, NJ, U.S.). Cell status was analyzed by dual staining method with Annexin V–FITC and PI, allowing the discrimination of necrotic (FITC^+^/PI^+^), apoptotic (FITC^+^/PI^−^), late apoptotic, or viable cells (FITC^−^/PI^−^). 

### 5.6. qRT-PCR Analysis of Cytokine Genes

IPEC-J2 cells were seeded with a density of 3 × 10^5^ cells per well in 6-well culture plates (Costar, Corning, NY, U.S.) and allowed to adhere for 24 h. Then, the cells were washed with PBS and treated with AFB_1_ and/or ZEA in serum-free medium for 24 h. Total RNA was extracted by using Trizol (Invitrogen, Carlsbad, CA, U.S.) according to the manufacturer’s instructions. The RNA pellets were dissolved in 30 μL RNase-free water and stored at −80 °C. The RNA concentration was determined using a NanoDrop ND-1000 Spectrophotometer (Nano-Drop Technologies, Wilmington, DE, U.S.) with purity ascertained (A260/A280) between 1.8 and 2.1. About 1 μg total RNA from each sample was converted into cDNA by using TB GREEN (TaKaRa, Dalin, China) for qRT-PCR analysis according to the manufacturer’s instruction. The qPCR was performed to quantify the target genes, such as *IL6*, *caspase-3*, tight junction (TJ) proteins (*occludin* and zonula occludens 1 (*ZO-1*)), and the others (see Table 3). The glyceraldehyde-3-phosphate dehydrogenase (*GAPDH*) gene was used as the housekeeping gene. The relative changes in gene expression levels of cytokines in IPEC-J2 cells normalized against *GAPDH* in response to mycotoxin treatments were determined by using the 2^−ΔΔCT^ method [52]. 

### 5.7. Data Analyses

All experiments were conducted with six replicates for each treatment. Data are presented as means ± SD, and one-way analysis of variance (ANOVA) with SPSS 20.0 (Sishu software (Shanghai) Co., Ltd. Shanghai, China) was applied. The Duncan test was used in this experiment. Differences were considered statistically significant at *p* < 0.05.

## Figures and Tables

**Table 1:** Dose time interacting effect of mycotoxins on relative cell viability (%). CFSCP: Cell-free supernatant of compound probiotics; MDE: Mycotoxin degradation enzyme.

Groups	6 h	12 h	18 h	24 h	48 h
Z500	83.48 ± 4.26Cb	101.79 ± 6.60Aa	94.18 ± 6.39Bab	90.15 ± 6.21Bc	103.72 ± 3.35Aa
A40	82.83 ± 3.48Cbc	95.04 ± 3.04Bb	94.94 ± 3.98Bab	97.72 ± 2.35Ba	103.48 ± 3.33Aa
Z500 + A40	81.02 ± 4.02Ccd	85.70 ± 5.77BCc	87.24 ± 6.62BCcd	88.63 ± 4.27ABbc	94.56 ± 4.21Abc
Z1000	79.40 ± 3.67Bcd	92.02 ± 5.61Ab	93.50 ± 3.69Aab	91.45 ± 6.47Ab	94.91 ± 5.90Abc
A80	81.58 ± 3.37Cbc	92.30 ± 3.21ABb	91.73 ± 3.63Bbc	95.84 ± 4.66ABa	97.27 ± 5.90Ab
Z1000 + A80	75.45 ± 5.81Cd	85.35 ± 7.28ABc	83.99 ± 4.44Bd	81.64 ± 2.72Bd	90.96 ± 3.78Ac
CFSCP + MDE	98.56 ± 0.45Aa	99.12 ± 0.67Aa	98.36 ± 0.06Aa	98.96 ± 0.43Aa	98.15 ± 0.55Ab

Note: Data with different lowercase letters in the same column significantly differ from each other (*p* < 0.05), while data with the same lowercase letter in the same column insignificantly differ from each other (*p* > 0.05). Data with different capital letters in the same row significantly differ from each other (*p* < 0.05), while data with the same capital letter in the same row insignificantly differ from each other (*p* > 0.05). The relative viability of IPEC-J2 cells (%) = (A490 in treated group - A630 in treated group)/(A490 in control group - A630 in control group) × 100. Z500 and Z1000 mean 500 and 1000 µg/L zearalenone (ZEA); A40 and A80 mean 40 and 80 µg/L aflatoxin B_1_ (AFB_1_).

**Table 2:** Effects of CFSCP + MDE on cell status induced by AFB_1_ and ZEA (%).

Groups	Q1	Q2	Q3	Q4
Control group	1.66 ± 0.09d	0.96 ± 0.24b	0.75 ± 0.30c	96.63 ± 0.38a
CFSCP + MDE	2.44 ± 0.24c	2.44 ± 0.42a	2.14 ± 0.36b	92.97 ± 1.00b
Z500	5.36 ± 0.45a	0.90 ± 0.39b	0.89 ± 0.14c	92.86 ± 0.56b
A40	4.19 ± 0.30b	2.33 ± 0.37a	2.94 ± 0.09a	90.54 ± 0.57c
Z500 + A40	5.13 ± 0.84a	2.85 ± 0.68a	2.50 ± 0.25b	89.52 ± 0.09c
Z500 + A40 + CFSCP + MDE	2.94 ± 0.32c	2.56 ± 0.43a	2.20 ± 0.25b	92.30 ± 1.00b

Note: Data with different lowercase letters in the same column significantly differ from each other (*p* < 0.05), while data with the same lowercase letter in the same column insignificantly differ from each other (*p* > 0.05). Q1, Q2, Q3, and Q4 represent necrotic, late apoptotic, early apoptotic, and viable cell rates, respectively.

**Table 3:** Primer sequences of some genes for RT-PCR.

Gene	Primer Sequence (5′-3′)	Accession Number	Size (bp)
*GAPDH*	F: ATGACCACAGTCCATGCCATC	XM-004387206.1	271
	R: CCTGCTTCACCACCTTCTTG		
Cell apoptosis genes			
*Bcl-2*	F: AGAGCCGTTTCGTCCCTTTC	XM-003122573.2	270
	R: GCACGTTTCCTAGCGAGCAT		
*Bax*	F: ATGATCGCAGCCGTGGACACG	XM-003355975.1	296
	R: ACGAAGATGGTCACCGTCTGC		
*Caspase-3*	F:TTGGACTGTGGGATTGAGACG	NM-214131.1	165
	R: CGCTGCACAAAGTGACTGGA		
Cytokines gene			
*IL-6*	F: GCTCTCTGTGAGGCTGCAGTTC	NM_213867.1	107
	R: AAGGTGTGGAATGCGTATTTATGC		
Barrier function genes			
*ZO-1*	F: CCTGAGTTTGATAGTGGCGTTGA	XM-003353439.2	269
	R: AAATAGATTTCCTGCCCAATTCC		
*Occludin*	F: ACCCAGCAACGACATA	NM_001163647.2	155
	R: TCACGATAACGAGCATA		
Nutrient transporter genes			
*SGLT1*	F: TCATCATCGTCCTGGTCGTCTC	M34044.1	144
	R: CTTCTGGGGCTTCTTGAATGTC		
*GLUT2*	F: ATTGTCACAGGCATTCTTGTTAGTCA	NM_001097417.1	273
	R: TTCACTTGATGCTTCTTCCCTTTC		
*PepT1*	F: CAGACTTCGACCACAACGGA	NM_214347.1	99
	R: TTATCCCGCCAGTACCCAGA		
*ASCT2*	F: CTGGTCTCCTGGATCATGTGG	DQ231578.1	172
	R: CAGGAAGCGGTAGGGGTTTT		
